# Negative Refraction Guided by a Glide-Reflection Symmetric Crystal Interface

**DOI:** 10.3390/ma18061210

**Published:** 2025-03-08

**Authors:** Yuzhong Zhou, Tian Sang, Yueke Wang

**Affiliations:** Optoelectronic Engineering and Technology Research Center, Jiangnan University, Wuxi 214122, China; 6221201012@stu.jiangnan.edu.cn (Y.Z.); sangt@jiangnan.edu.cn (T.S.)

**Keywords:** phononic crystal, negative refraction, glide-reflection symmetry

## Abstract

Research on phononic crystals with negative refractive indices constitutes the most crucial approach to achieving ultra-high-resolution acoustic lenses. This study presents a glide-reflection (GR) symmetrical phononic crystal (PC), and the mismatch of the Wannier center between two PCs leads to the emergence of edge states (ESs). By constructing a single-domain wall, the negative refraction is achieved due to the excitation of ESs with negative dispersion. Further, by stacking multiple GR symmetric PC interfaces, the coupled edge states (CESs) are found, which originate from the coupling between the adjacent interfaces. Thus, stronger negative sound refraction effects with negative transverse displacement can be achieved, because the incident sound wave can be coupled into the CESs with negative dispersion. Simulation results are conducted using the finite element method to verify our idea, and our research provides a novel methodology for the design of acoustic negative refraction.

## 1. Introduction

Since the integration of topological concepts into physics in 1980 [1,2], topological insulators have emerged as a prominent research field in condensed matter physics. These materials have garnered significant attention for their distinctive properties, especially in manipulating electron transport for information or energy transfer. By creating topological heterostructures through modifications in crystal structures, applying external fields or doping, topological edge states can be generated [3,4,5]. These edge states exhibit remarkable characteristics such as robustness against disorder and immunity to backscattering [6,7,8], which greatly enhance transport efficiency. In recent years, the study of topological insulators has expanded beyond conventional condensed matter systems to encompass other classical wave systems such as photonic [9,10,11], mechanical [12,13], and phononic [13] systems. Inspired by topological insulators, topological phononic crystals (PCs) are often investigated for their applications in topological phononic waveguide. Well-known topological phononic insulators like acoustic quantum spin Hall insulators and acoustic valley Hall insulators enable one-way wave propagation, that is immune to backscattering, along domain walls composed of PCs with opposite topological phases [14,15,16,17,18].

Negative refraction was first theoretically proposed by Veselago in 1968 [19], who predicted that when both the permittivity ϵ and permeability μ of a metamaterial are simultaneously negative, electromagnetic waves would exhibit a phase velocity opposite to the direction of energy flow, leading to negative refraction. This concept was later extended to acoustics, where researchers demonstrated that negative refraction of sound waves could be achieved by designing specific acoustic metamaterial structures [20]. Unlike electromagnetic waves in optical systems, the propagation of acoustic waves depends solely on the elastic properties of the medium. As a result, achieving negative refraction in acoustic metamaterials typically relies on local resonances or structural dispersion effects to manipulate the effective density or modulus [21,22]. However, these approaches often require precise tuning of effective parameters and are constrained by factors such as material loss, fabrication accuracy, and operational bandwidth [23].

In recent years, phononic crystals have emerged as a promising alternative for achieving negative refraction, owing to their periodic structures [24,25,26]. Unlike metamaterials, which rely on modulation of effective parameters [27,28], phononic crystals leverage their topological band structure to enable negative refraction [29]. Moreover, recent advancements in topological acoustics have shown that topologically protected edge states can be harnessed for negative refraction, providing unique advantages for the manipulation of acoustic waves [30]. When compared to traditional negative-refraction metamaterials, the approach based on topologically protected edge states presents several key benefits: it facilitates negative refraction across a broader frequency range and wider incident angles, extends material compatibility, and enhances both tunability and robustness [31].

In this paper, we introduce a novel method that utilizes a glide-reflection (GR) symmetrical PC for achieving negative refraction. The GR dislocation creates edge states, which can be found along the interface between two PCs whose Wannier centers are not the same as each other [32]. Due to the excitation of the ES with negative dispersion, negative refraction is achieved in the single-domain wall structure. Furthermore, coupled edge states (CESs) can be excited in multiple-domain walls, originating from the coupling between adjacent interfaces. Larger negative transverse displacement and stronger negative refraction can be observed when the incident acoustic wave excites the CESs with negative dispersion. All the verification is conducted by the finite element method (FEM), and the results open up new avenues for manipulating acoustic waves, with potential applications in super-resolution imaging and wave communication.

## 2. Numerical Results and Discussion

**Figure 1:**
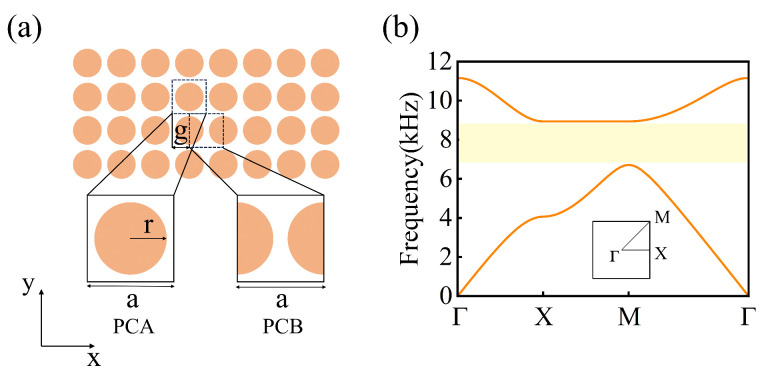
(**a**) Schematic of the two-dimensional PCs composed of epoxy cylindrical scatterers arranged in an air background; and the insets are the choices of unit cell (PCA and PCB). (**b**) The phononic band structure for the proposed PCs, the yellow area represents a complete band gap Inset: The first Brillouin zone.

Our study starts with a square lattice composed of epoxy cylindrical scatterers placed in an air background. Figure 1a shows the proposed PC, and the radius of each scatterer is *r* (=0.416*a*), where *a* (=2.5 cm) represents the lattice constant. We take the unit cell containing a cylindrical scatterer as the original unit, defined as PCA, as shown in the lower-left part of Figure 1a. Another choice of unit cell for PC is also shown in the lower-right part of Figure 1a, where two half-cylindrical scatterers are evenly distributed on the left and right sides of the unit cell, defined as PCB. All numerical simulations in this study were conducted using COMSOL Multiphysics 6.1 based on the finite element method. Figure 1b presents the phonon band structure of PCA(PCB), where a complete band gap is observed between the first and second bands, indicated by a yellow region. The complete band gap between 6556 Hz and 9045 Hz arises from Bragg scattering within the periodic structure [33]. When sound waves propagate through a medium with a periodic structure, a periodic arrangement of scatterers induces strong multiple scattering. The interference between these scattered waves leads to destructive interference within specific frequency ranges, blocking the propagation of sound waves and forming band gaps. Here, we define *g* as the gliding parameter along the *x*-direction, as shown in Figure 1a. Glide reflection (GR) refers to a symmetry operation in which a crystal reflects within a plane and then moves a certain distance along that plane. This translation distance is the gliding parameter, which specifically determines the displacement of the crystal in the x-direction. Therefore, PCA (PCB) can be achieved by gliding PCB (PCA) by *g* (=a/2). PCA and PCB possess different topological properties, due to glide-reflection (GR) symmetric dislocations.

**Figure 2:**
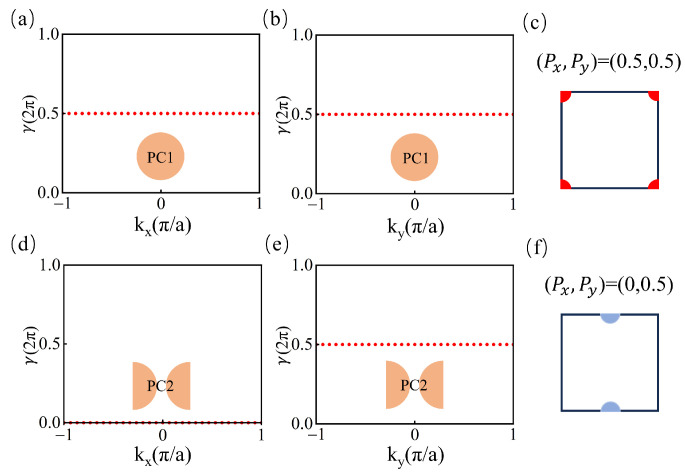
(**a**,**b**) The Berry phase $\gamma_{y,k_{x}}\left(\gamma_{x,k_{y}}\right)$ as a function of $k_{x}$ ($k_{y}$) for PCA, indicating nontrivial bulk polarization $\left(P_{x},P_{y}\right)=\left(0.5,0.5\right)$. (**c**) The configurations of primitive cell PCA and its Wannier configurations. (**d**,**e**) The Berry phase $\gamma_{y,k_{x}}\left(\gamma_{x,k_{y}}\right)$ as a function of $k_{x}$ ($k_{y}$) for PCB, indicating nontrivial bulk polarization $\left(P_{x},P_{y}\right)=\left(0,0.5\right)$. (**f**) The configurations of primitive cell PCB and its Wannier configurations.

PCA has C4 symmetry, whereas PCB has C2 symmetry. According to the International Tables for Crystallography, the Wyckoff positions [34] for crystals with twofold rotation symmetry are located at the center, corners, and midpoints of edges. Using the Wilson-loop method, we are able to calculate the Berry phase to determine the bulk polarization parameter and identify the position of the Wannier center. Figure 2 illustrates the Berry phase $\gamma_{y,k_{x}}\left(\gamma_{x,k_{y}}\right)$ for the first band along the closed loop $k_{x}$ ($k_{y}$) for a fixed $k_{y}$ ($k_{x}$). For PCA, as depicted in Figure 2a,b, the Berry phase γ along both $k_{x}$ and $k_{y}$ is 0.5, indicating nontrivial bulk polarization $\left(P_{x},P_{y}\right)=\left(0.5,0.5\right)$. Consequently, the Wannier center of the first band is located at the four corners of the unit cell, as shown in Figure 2c. Similarly, for PCB, as shown in Figure 2d,e, the Berry phase along $k_{x}$ is 0, and along $k_{y}$ is 0.5, indicating nontrivial bulk polarization $\left(P_{x},P_{y}\right)=\left(0,0.5\right)$. The Wannier center is located at the midpoints of the top and bottom edges, as illustrated in Figure 2f. Hence, we can obtain two different Wannier configurations: one is the PCA, whose symmetric Wannier functions are centered at the corners (denoted by red); and the other is the PCB, whose symmetric Wannier functions are centered at the midpoints of edges (denoted by blue). The discrepancy in the Wannier centers between PCA and PCB leads to the emergence of edge states along the interface between PCA and PCB.

**Figure 3:**
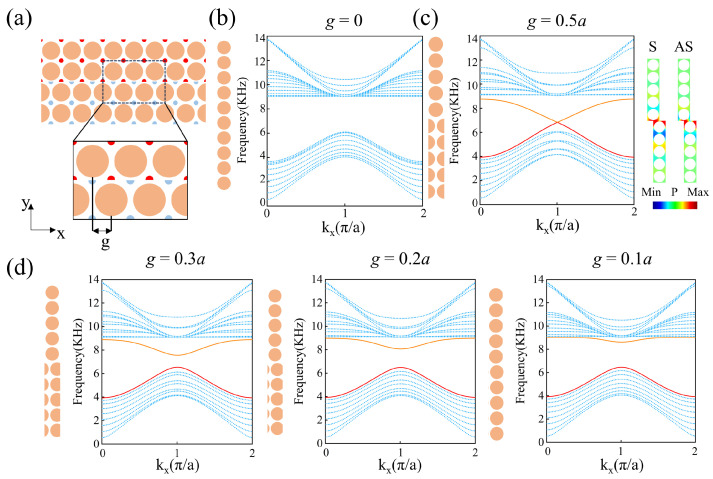
(**a**) The phononic crystal heterostructure (PCA/PCB) with a single-domain wall; (**b**) the projected phonon band when gliding parameter g=0. (**c**) Left panel: the projected phonon band with g=0.5a, where the red and orange lines refer to a pair of edge states in the band gap. Right panel: the modal distributions of the S and AS states for $k_{x}a/\pi =0.8$. (**d**) The projected phonon bands for g=0.3a, g=0.2a, and g=0.1a from left to right.

We constructed a topological phononic crystal heterostructure with a single-domain wall using PCA (upper) and PCB (lower), as shown in Figure 3a. The red and blue dots on the PCs represent different positions of the Wannier center. Next, we calculated the projected bands with g=0 and g=a/2 in our system, as shown in Figure 3b and 3c, respectively. The left panels of Figure 3b,c are the supercell with g=0 and g=a/2, respectively. Periodic boundary conditions are applied on the vertical boundaries of the supercell while the horizontal boundaries are left free. And the right panels display the corresponding projected band structures. Figure 3b demonstrates a complete band gap similar to that observed in Figure 1b, while the bulk bands are characterized by blue dotted lines. Figure 3c shows the projected band structure of the domain wall when g=0.5a. The GR symmetry at the interface ensures the presence of a pair of edge states in the band gap, which are plotted with red and orange lines. The two edge states are degenerate at $k_{x}=\pi /a$ (*X* point of the Brillouin zone), leading to the presence of a Dirac point. The pressure field distributions for the lower and upper branches of the edge states at $k_{x}a/\pi =0.8$ are shown on the right side of Figure 3c. The pressure-field pattern of the lower (upper) branch is symmetric (antisymmetric), and we denote the edge state of the lower (upper) branch as the S(AS) state. To investigate the effects of the gliding parameter *g* on the edge states, we calculated the projected bands for different values of *g*, as shown in Figure 3d. From left to right, the band projections are plotted for g=0.3a, g=0.2a, and g=0.1a. When *g* deviates from 0.5a, the degeneracy of the two edge states at the *X* point vanishes, resulting in a complete band gap. The width of the band gap increases as *g* decreases. Therefore, the dispersion of the two edge states can be controlled by the gliding parameter *g*. The upper branch of the edge states possess the negative slope, which provides a possible way of achieving negative refraction.

**Figure 4:**
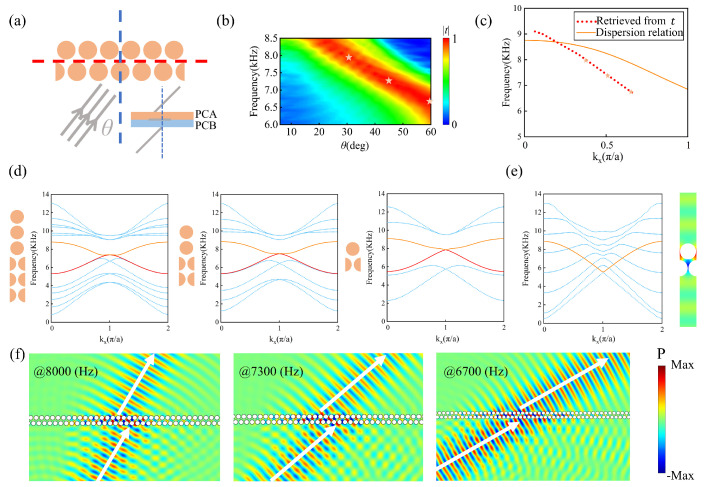
(**a**) Schematic drawing of a plane wave incident on a single-domain wall, the red dashed line represents the edge state, and the blue dashed line represents the normal of the incident wave. Inset: diagrammatic sketch of negative refraction. (**b**) The transmission spectra as functions of incidence angle θ and frequency *f*. The color represents the transmission amplitude. (**c**) The dispersion retrieved from the transmission spectra (red dots) and the dispersion of Figure 3c (orange solid curves). (**d**) The projected phonon bands when N = 6, 4, 2. As the number of layers N decreases, the boundary states remain stable. (**e**) Left panel: the projected phonon band with air layer. Right panel: the modal distributions of the upper boundary state (the solid curves) are shown on the right for $k_{x}a/\pi =0$. (**f**) The simulation results of the negative refraction effects, for incident angles of $30^{{}^{\circ}}$, $45^{{}^{\circ}}$, and $60^{{}^{\circ}}$ from left to right. The white arrows indicate the direction of energy flow.

Then, we utilize the upper branch of the edge state with g=0.5a to achieve negative refraction. As illustrated in Figure 3c, the edge state of the upper branch exhibits negative dispersion in a broad range of $k_{x}$, indicating that negative refraction can be achieved over a wide range of incident angles. The proposed phononic crystal interface with GR symmetry can be used to manipulate sound waves propagating in the direction opposite to conventional refraction, thereby enabling novel wave manipulation. To validate this idea, we conducted simulations for the transmission of sound waves when a plane wave is incident on a single-domain wall structure with g=0.5a, as illustrated in Figure 4a, where the gray line represents the incident plane wave at an angle of θ. Here, there is only one layer of PCA (PCB), which makes it easy to couple the incident plane wave into the edge state. The blue dashed line represents the normal of the interface, while the red dashed line represents the boundary between PCA and PCB. We selected the frequency (ranging from 6 kHz to 8.5 kHz) and incident angle (ranging from $8^{{}^{\circ}}$ to $60^{{}^{\circ}}$) of the incident sound wave as parameters to evaluate the transmission coefficient t(f,θ), as shown in Figure 4b. It is observable that over a broad range of incident angles, there is a specific frequency range that yields a high transmission coefficient, which originates from the excitation of the edge states. Moreover, owing to the conservation of momentum (wave vector) along the interface direction, we can deduce the dispersion relationship from the transmission spectrum and compare it with the dispersion in Figure 3c. In Figure 4c, the dispersion relationship retrieved from the transmission spectrum is plotted with red dots, and the dispersion in Figure 3c is plotted with an orange solid line. The similarity between the two dispersion relationships indicates that our designed structure can achieve effective coupling between the incident sound wave and the edge states. The observed discrepancy between the two dispersion relationships can be attributed to the influence of the air layer. In the previous simulation of band projection, we added a sufficient number of PCs (N = 10) on both sides of the structure to prevent interference from the air layer, providing a complete boundary band dispersion distribution without band interference, thereby facilitating the analysis of subsequent phenomena. However, in the subsequent simulation of negative dispersion, to meet the needs of practical negative refraction structure design and increase the transmittance of incident waves, it is necessary to minimize the absorption of waves by the boundaries as much as possible, which requires reducing the number N of unit cells in the vertical direction. We plotted the band projection as the number of layers N of the supercell strip gradually decreased, as depicted in Figure 4d. It is evident that as the number of layers N decreases, the boundary states persist stably. Even when the strip comprises only one unit cell each of PCA and PCB (N = 2), the boundary states remain intact. So that, in the case of N = 2, it can be considered that an infinitely long air layer is located at the top and bottom of the system, and the ineffective band in the air layer will enter the band gap, resulting in a faster descent of the boundary band along the positive kx direction and a decrease in the frequency of degenerate Dirac points. The new boundary band generated in this way is quite consistent with the dispersion retrieved from the transmission spectra, as shown in Figure 4e.

Simultaneously, to validate the negative refraction behavior of sound waves, we simulated the pressure field generated by a plane wave incident on a single-domain wall phononic crystal structure, as illustrated in Figure 4f. The upper boundary branch of the edge states exhibits negative dispersion, leading to the transmission of waves along the interface in the negative direction of the wave vector. When the incident sound waves are coupled into the edge states, a negative displacement is induced at the boundary, resulting in the outgoing sound wave at the far side being emitted on the same side of the incident wave, thereby producing the effect of negative refraction. Specifically, we chose incident angles of $30^{{}^{\circ}}$, $45^{{}^{\circ}}$, and $60^{{}^{\circ}}$, corresponding to frequencies of 8000 Hz, 7300 Hz, and 6700 Hz (denoted by the yellow stars in Figure 4b), to simulate the negative refraction effect, as illustrated in Figure 4f. It is observed that the exit position of the outgoing wave beam is on the left side of the normal, achieving negative refraction. Additionally, due to the mismatch between the phononic crystal and air, there is reflection on the lower surface, with the reflection angle approximately equal to the incident angle, consistent with the conventional reflection law. Furthermore, it can be seen that the incident wave experiences a displacement negative to the wave-vector component along the interface. In the single-domain wall structure, the length of negative displacement is approximately 2a (=5 cm). Although relatively small compared to the overall structural dimensions, our design allows for tunable control of the negative displacement by constructing multiple-domain wall structures, enabling adjustments to meet practical device design requirements, as discussed in the following sections.

**Figure 5:**
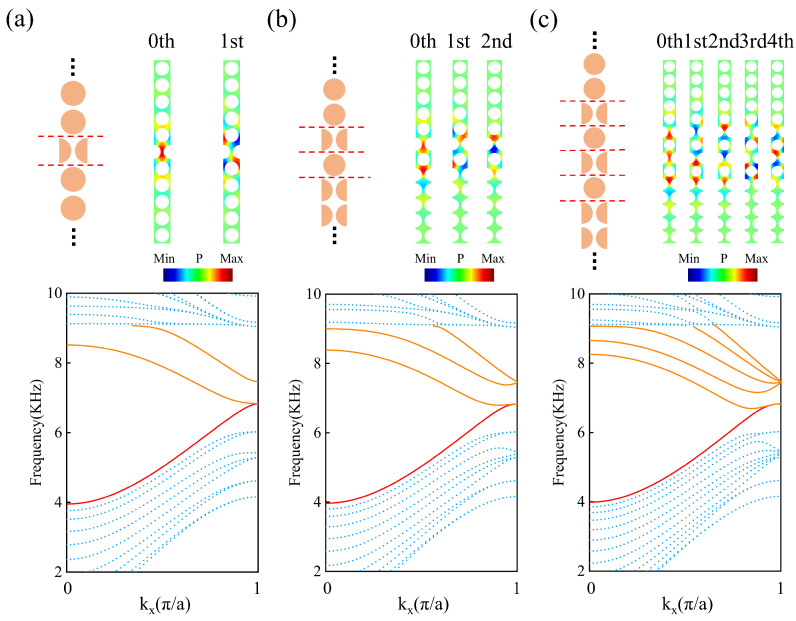
The supercell and phonon projected bands of 2-domain walls (**a**), 3-domain walls (**b**), and 5-domain walls (**c**). The modal distributions of CESs (denoted by orange solid curves) from low frequency to high frequency are shown in the upper-right parts of (**a**–**c**), for $k_{x}a/\pi =0.8$.

Further, we consider the multiple-domain walls based on GR symmetric phononic crystals, where the couplings between edge states and negative refraction occurs. The projected phonon bands for two-domain wall, three-domain wall, and five-domain wall structures were calculated, as shown in Figure 5. The upper-left parts of Figure 5a–c show the supercells with two-domain walls, three-domain walls, and five-domain walls, corresponding to PCA/PCB/PCA, PCA/PCB/PCA/PCB, and PCA/PCB/PCA/PCB/PCA/PCB structures. Here, the gliding parameter *g* for the adjacent PCs is 0.5a, and the red dashed lines indicate the interfaces between PCA and PCB. For multiple-domain wall structures, when the adjacent interfaces are close to each other, due to the coupling between the edge phonon states, coupled edge states (CESs) can be observed. The lower parts of Figure 5a–c show the projected phonon bands for two-domain walls, three-domain walls, and five-domain walls. Two, three, and five upper branches of the CESs are found, which are plotted with orange solid lines, corresponding to two-domain walls, three-domain walls, and five-domain walls, respectively. In a similar way, the lower branches of the CESs are also achieved, but one, two, and four lower branches of the CESs are involved in the bulk bands. Thus, only one lower branch of the CES is found in the band gap, which is plotted with a red solid line. In addition, the blue dots represent the bulk bands. For the two-domain wall, three-domain wall, and five-domain wall, the upper branches of the CESs exhibit negative slopes in a wide $k_{x}$ range, thus enabling the realization of negative refraction within a multiple-domain structure. It is observed that a common frequency range can be found where the upper branches of CESs are simultaneously in their negative dispersion regions. Furthermore, we calculated the modal distributions of CESs with negative dispersion when $k_{x}a/\pi =0.8$. In the upper-right parts of Figure 5a–c, the modal distributions are shown for the 0th and 1st; 0th, 1st, and 2nd; and 0th, 1st, 2nd, 3rd, and 4th modes, from low frequency to high frequency, respectively. The upper branches of CESs possess the negative slope, which provides a possible way of achieving negative refraction with controllable negative displacement.

**Figure 6:**
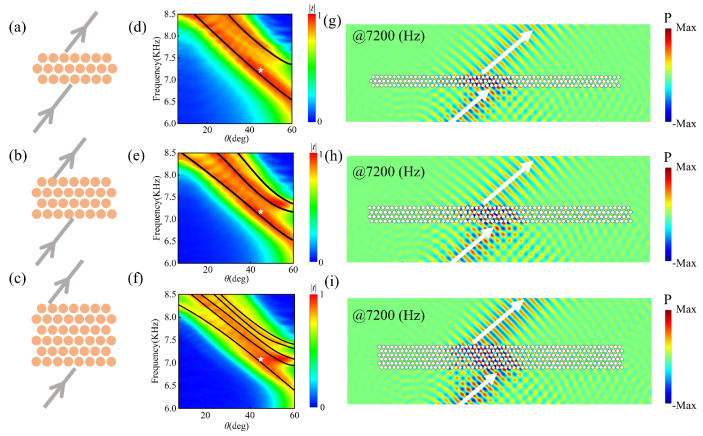
(**a**–**c**) Schematic of 2-domain wall (**a**), 3-domain wall (**b**), and 5-domain wall (**c**) structures supporting negative refraction. (**d**–**f**) The transmission spectra as functions of incidence angle θ and frequency *f* for the structures (**a**–**c**), respectively. (**g**–**i**) The simulation results of the negative refraction effects, for incident angle of $45^{{}^{\circ}}$ and frequency of 7200 Hz, for the structures (**a**–**c**), respectively. The white arrows indicate the directions of energy flow.

Similar to the scenario with a single-domain wall, we construct the air/PCA/PCB/PCA/air (two-domain wall), air/PCA/PCB/PCA/PCB/air (three-domain wall), and air/PCA/PCB/PCA/PCB/PCA/PCB/air (five-domain wall) structures, as shown in Figure 6a–c. We aim to achieve negative refraction, based on the upper branches of CESs with negative dispersion, and the arrows indicate the incident and refracted sound waves. The transmission coefficients were calculated separately when a plane wave was incident on multiple-domain structures, as shown in Figure 6d–f, corresponding to two-domain wall, three-domain wall, and five-domain wall structures. The parameter space is the same that of Figure 4b. The positions of the strongest transmission coefficients are plotted with black solid lines, which are in accordance with the dispersion of the upper branches of the CESs in Figure 5. It is verified that the multiple-domain wall structures can achieve effective coupling between the incident sound wave and the CESs.

Figure 6g–i illustrate the pressure distributions of two-domain walls, three-domain walls, and five-domain walls, when the frequency of the incident sound wave is 7200 Hz under a $45^{{}^{\circ}}$ incident angle, corresponding to the white star in Figure 6d–f. Notably, the outgoing sound waves exhibit a strong negative refraction effect, resulting in negative displacements of 6a (=15 cm), 9a (=22.5 cm), and 13a (=32.5 cm), corresponding to the cases of two-domain walls, three-domain walls, and five-domain walls, respectively. Moreover, the increasing distance of the outgoing sound waves from the normal with additional layers of domain walls, along with the layered patterns of the internal field within the structures, indicates that each layer of domain walls guides the sound waves with negative displacement. These results confirm that using GR symmetric phononic crystals to construct multiple-domain structures effectively creates a coupled waveguide array, facilitating the realization of negative refraction with a wide range and multiple degrees of control. Compared with the results of the single-wall structure in Figure 4, the multiple-domain walls can achieve negative refraction with large negative displacement. This approach offers a versatile method for exploring topological heterostructures capable of realizing negative refraction.

**Figure 7:**
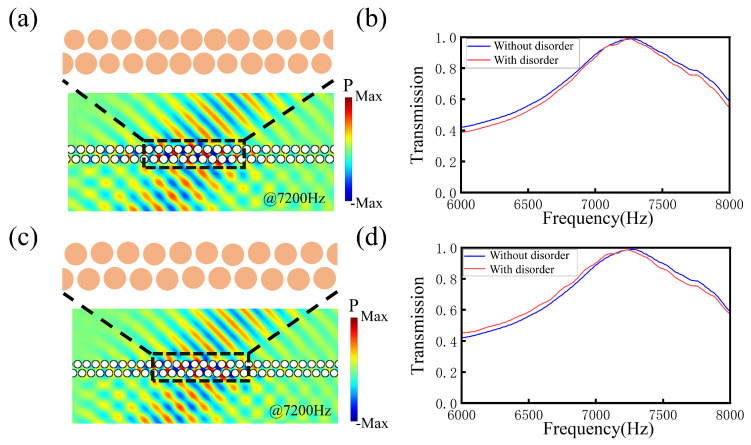
(**a**) Illustration of the single-domain wall structure with radius disorder. (**b**) Transmission curve of the structure without radius disorder (indicated by blue line) and with radius disorder (indicated by red line). (**c**) Illustration of the single-domain wall structure with position disorder. (**d**) Transmission curve of the structure without position disorder (indicated by blue line) and with radius disorder (indicated by red line).

To confirm the high fabrication tolerance of our design, we introduce disorder to the radius and position of the scatterers. Here, we take the single-domain wall structure in Figure 4 with an incident angle of $45^{{}^{\circ}}$, for example, and restrict the perturbations of the radius and position below the levels of 4% and 2%, respectively. As shown in Figure 7a, the region with radius disorder is indicated by the black dashed rectangle. Then, we calculate the transmission of the single-domain wall. As shown in Figure 7b, there is no difference for the structures with and without radius disorder, which shows that our structure is robust. Similarly, Figure 7c presents a schematic of the single-domain wall structure with position disorder. As shown in Figure 7d, there is no difference for the structures with and without position disorder, which also shows that our structure is robust.

## 3. Conclusions

In conclusion, we proposed a novel strategy to achieve negative refraction in GR symmetric PC, whose bulk topological index is characterized by the position of the Wannier center. In the single-domain wall structure, the edge state can be found due to the mismatch of the Wannier centers. Through FEM simulation, a negative refraction effect of the single-domain wall is verified, where the incident sound wave excites the ES with negative dispersion. Furthermore, we designed multiple-domain wall structures by stacking GR symmetric phononic crystals interfaces, achieving coupled edge states. Stronger negative refraction was found, where the incident sound waves incident on the multiple-domain wall excites the CESs with negative dispersion.

Compared to conventional metamaterial approaches, our approach enables negative refraction over a broad range of incident angles, with optimal operating frequencies precisely determined for different incident conditions. The design of multiple-domain wall structures allows for fine-tuning of the refraction behavior. By increasing or decreasing the number of stacked-domain walls, the exit position of the negatively refracted wave can be effectively controlled, enabling precise manipulation of bulk wave propagation. This adjustability provides additional flexibility for practical applications such as wave steering and signal routing.

To experimentally verify the acoustic negative refraction effect, a feasible approach is as follows. The proposed phononic crystal structure could be fabricated by 3D printing, and the base material could be epoxy. In the measurement process, the excitation acoustic signals would be controlled by two clock-synchronized arbitrary waveform generators through the pre-programmed LabVIEW software. The acoustic pressure field could be measured using a microphone mounted on a two-dimensional scanning system, allowing for point-by-point scanning across the entire region of interest. By interpolating the collected data and applying Fourier analysis, the full-field pressure distribution could be reconstructed, enabling a direct comparison with our numerical results. Furthermore, by extracting key parameters such as transmission and refraction angles, the negative refraction effect could be quantitatively assessed.

## 4. Highlight

This study proposes a glide-reflection symmetric phononic crystal enabling negative refraction via edge states. Multiple-domain walls enhance the refraction strength and offer tunable wave control. The design imposes no strict material constraints and supports broad-angle operation, improving practical applicability.

## Data Availability

The original contributions presented in this study are included in the article. Further inquiries can be directed to the corresponding author.

## Abbreviations

The following abbreviations are used in this manuscript:

GR	Glide reflection
PC	Phononic crystal
ES	Edge state
CES	Coupled edge state
FEM	Finite element method
WPs	Wyckoff positions
